# Acute cyclosporine overdose in a child with nephrotic syndrome: a case report and literature review

**DOI:** 10.3389/fped.2026.1737399

**Published:** 2026-01-22

**Authors:** Eun Song Song, Eun Mi Yang

**Affiliations:** Department of Pediatrics, Chonnam National University Hospital and Medical School, Gwangju, Republic of Korea

**Keywords:** acute toxicity, children, cyclosporine, nephrotic syndrome, treatment

## Abstract

**Background:**

Calcineurin inhibitors are widely used in organ transplantation and nephrotic syndrome, with chronic toxicities well documented, but acute toxicity is rarely reported.

**Case presentation:**

We report a 3.9-year-old boy with steroid-dependent nephrotic syndrome who accidentally ingested a single dose of cyclosporine A (CsA) at 75 mg/kg (1,500 mg)— over 20 times the intended dose of 3.5 mg/kg (70 mg)—due to medication error. He developed transient abdominal pain, diarrhea, and vomiting, which resolved without treatment. Error was discovered 12 h post-ingestion, and he was hospitalized with a CsA trough level of 1003 ng/mL (measured 13 h after ingestion), yet remained clinically stable. Management included CsA discontinuation, intravenous hydration, rifampin- and phenobarbital-induced cytochrome P450 activation, resulting in normalization of CsA levels within 48 h. A literature review identified 28 pediatric cases of acute CsA overdose, with presentations ranging from asymptomatic to severe neurotoxicity and acute kidney injury. The varied, including supportive care, gastrointestinal decontamination, and enzyme induction, with generally favorable outcomes.

**Conclusions:**

Although rare, acute CsA overdose in children can pose serious risks. This case and review underscore the symptoms of overdose and prompt intervention to prevent complications.

## Introduction

Cyclosporine A (CsA), a potent calcineurin inhibitor, is a widely used immunosuppressant the revolutionized organ transplantation and remains a cornerstone in treating pediatric nephrotic syndrome. Due to its narrow therapeutic index, CsA requires regular drug level monitoring and vigilance for adverse effects, particularly chronic toxicities such as nephrotoxicity and neurotoxicity, which are well documented. In contrast, acute CsA overdose, especially in children, is poorly understood.

Pediatric drug ingestion is a major cause of emergency visits, with accidental ingestion common in young children and intentional overdose more frequent among adolescents (1). Acute CsA toxicity presents variably, from asymptomatic to severe, influenced by age, administration route, concurrent use of nephrotoxic drugs, and comorbidities (2). Children, especially premature infants, are more vulnerable, and parenteral overdose carries high morbidity and mortality, often necessitating urgent interventions (2). Symptoms may include headache, nausea, vomiting, hypertension, and in severe cases, kidney injury, tachycardia, hepatitis, seizure (status epilepticus), or death (3). Therapeutic strategies are inconsistently reported but typically involve drug discontinuation, hospitalization, and supportive measurement such as gastric lavage, activated charcoal, and cytochrome 450 enzyme induction. In severe cases, hemodialysis or whole blood exchange may be required (4). We report a case of accidental cyclosporine overdose in a child with nephrotic syndrome, caused by a caregiver error. Despite a markedly elevated cyclosporine level, the patient recovered uneventfully after treatment with enzyme inducers. This case underscores the importance of careful medication administration and reviews current management strategies for acute cyclosporine toxicity.

## Case presentation

**Figure 1:**
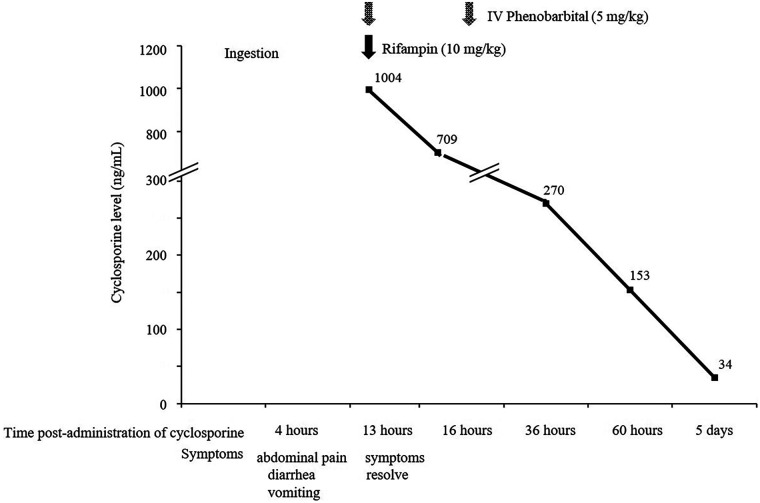
Clinical course of cyclosporine levels, therapeutic interventions, and symptoms.

A 3-year-old boy, diagnosed with nephrotic syndrome at age 2, was referred for frequent relapses. He was diagnosed with steroid-dependent nephrotic syndrome and started on oral CsA. While receiving 70 mg (3.7 mg/kg) twice daily, his outpatient CsA trough levels remained between 61 and 64 ng/mL, and he was relapse-free. Due to a medication error, his grandmother mistakenly administered a 21.4-fold overdose (1,500 mg), confusing CsA with a cold remedy. The dosing error was discovered by his mother 12 h later, before the next scheduled dose, after she noticed the medication bottle was markedly emptier than expected. At dawn, the patient experienced transient gastrointestinal symptoms including abdominal pain, diarrhea, and vomiting. These symptoms emerged during the expected peak early exposure window, which typically occurs within 2–4 h post-administration in children according to previous pharmacokinetic studies. The symptoms resolved spontaneously by morning, and the patient remained asymptomatic at the time of discovery. Consequently, the mother initially questioned the need for a hospital visit, as she was unaware that such a massive overdose could result in severe clinical complications. However, after consulting with the hospital and being advised to seek immediate evaluation, she brought the child to the hospital. On arrival, the patient was clinically stable and no subjective symptoms were reported by himself. A detailed physical examination revealed no abnormalities; his abdomen was soft and non-tender without organomegaly, and his neurological status was unremarkable. Vital signs were within normal limits for his age; blood pressure 102/63 mmHg, heart rate 105 beats/min, and respiratory rate 24 breaths/min. Laboratory studies were largely unremarkable, with the following details: white blood cell count, 13,700/μL; hemoglobin, 11.6 g/dL; platelet count, 447,000/μL; serum sodium, 138 mEq/L; serum potassium, 4.4 mEq/L; serum chloride, 106 mEq/L; serum calcium, 10.0 mg/dL; serum phosphorus, 4.8 mg/dL; blood urea nitrogen, 14.8 mg/dL; serum creatinine, 0.26 mg/dL; serum total protein, 6.3 g/dL; serum albumin, 4.3 g/dL; serum alanine transaminase, 24 U/L; and C-reactive protein, 0.06 mg/dL. The serum lactate level was mildly elevated [2.4 mmol/L (normal range 0.5–2.2 mmol/L)] and normalized within 12 h. Plasma CsA concentration measured 13 h post-ingestion was 1,004 ng/mL. The diagnosis of acute CsA overdose was confirmed by correlating the patient's clinical course with pharmacological data. The rapid resolution of symptoms, combined with the absence of fever or elevated inflammatory marker, allowed for the exclusion of alternative etiologies such as acute infection or an exacerbation of nephrotic syndrome. Furthermore, the markedly elevated plasma CsA concentration, measured 13 h after the reported ingestion, provided definitive objective evidence of a massive acute overdose, even with the delayed hospital presentation. Active charcoal was withheld due to the delay. CsA was discontinued, and intravenous hydration was initiated. Rifampin (10 mg/kg) and intravenous phenobarbital (5 mg/kg every 12 h for 24 h) was administered. He remained asymptomatic with no neurological deficits. Vital signs were stable, and he maintained normal urine output. Repeated laboratory tests, including complete blood count and kidney and liver function tests, remained within normal limits. He was monitored for 36 h; CsA concentration decreased from 1,004 ng/mL to 153 ng/mL within 49 h (Figure 1). After 3 days, the CsA concentration had fallen below 50 ng/mL, and oral CsA was restarted at the regular dose. The patient has since had an uneventful clinical course with normal serum creatinine levels.

## Discussion

Although CsA overdose has been reported in transplant and nephrotic syndrome patients, pediatric cases are less commonly reported. This case describes an accidental acute CsA overdose in children with nephrotic syndrome and the highlights management using enzyme inducers.

CsA, a calcineurin inhibitor (CNI), suppresses immune response by downregulating cytokine gene transcription. It is primarily used to prevent rejection solid organ and bone marrow transplantation and to treat autoimmune and inflammatory conditions, including psoriasis, atopic dermatitis, Crohn's disease, and notably, nephrotic syndrome (5–7). CNIs undergo extensive intestinal and hepatic metabolism via cytochrome P450 enzyme system (CYP) 3A4 (8). Therefore, acute overdose can occur not only form dosing errors but also from CYP downregulation during sepsis, graft failure, or drug interaction (9, 10). Drugs that increase CsA levels include erythromycin, clarithromycin, ketoconazole, voriconazole, itraconazole, corticosteroids, calcium channel blockers, and sex hormones (11, 12). Approximately 80% of CsA overdoses occur in organ transplant patients (2), reflecting its widespread use and frequent co-administration with interacting drugs. In nephrotic syndrome, a recent pediatric case report indicated that CsA overdose was caused by prescription and administration errors (3), as in our case involving caregiver misadministration. Medication errors in children are common yet preventable causes of patient harm, occurring at various stages from prescribing to drug administration. As in our case, most important factor in preventing medication error by caregiver is thorough and repetitive education, with a strong emphasis on precise dosing techniques to ensure patients safety and avoid unnecessary harm.

In most acute CsA overdose cases, symptoms of toxicity are often mild and can be managed solely with supportive care (3, 13, 14). Clinical manifestations appear to be dose-dependent; symptomatic cases involve a significantly higher intake (20.8 ± 28.8 times the usual dose) compared to asymptomatic ones (4.4 ± 3.4 times) (15). While enteral overdoses are generally well-tolerated, intravenous overdoses are poorly tolerated and can be fatal (15). According to a recent systematic review, pediatric patients account for 29.2% of all CsA overdose cases (2). Since the required volume of liquid formulation is generally small, even a 20-fold error may result in a total volume that still appears reasonable to an inexperienced caregiver. This lack of visual disparity makes such medication errors particularly difficult to detect during administration, thereby increasing the risk of significant accidental toxicity (5). Although children tend to metabolize CsA more rapidly and hence require higher dosages to achieve therapeutic levels, they can still be more vulnerable to toxicity as a result of variations in drug absorption, clearance, and tissue susceptibility (16). Acute CsA overdose in children presents as mild to severe, depending on several risk factors. According to recent epidemiological data, gastrointestinal (GI) disturbances are the third most frequent manifestation of CNI intoxication (14.6%), following nephrotoxicity (46.3%) and neurotoxicity (22.0%) (12). Consistently, our review of 29 pediatric cases, including the present case, revealed a broad clinical spectrum: while seven cases remained asymptomatic, the remainder exhibited diverse symptoms ranging from gastrointestinal symptoms (nausea and vomiting) and central nervous system (CNS) manifestations (seizure and coma) to nephrotoxicity and cardiovascular changes, such as hyponatremia, renal failure, and blood pressure fluctuations (Table 1). Cyclosporine toxicity is influenced by multiple risk factors, including young age (particularly prematurity), parenteral administration, and concurrent use of nephrotoxic drugs, and underlying conditions such as prematurity, low birth weight, infection, and renal or respiratory failure (2, 3). One fatal case involved several of these risk factors, including age, parenteral administration, and prematurity (4). Another patient who developed epilepsy had a history of febrile seizures, a known risk factor for neurologic impairments (17). Although no definitive serum concentration threshold for symptom onset has been established, a recent systematic review suggests that oral dose exceeding 400 mg/kg or serum levels above 3,800 ng/mL may be fatal. Seizures have been associated with levels above 500 ng/mL, and coma with levels exceeding 1,000 ng/mL (2). In our case, despite a serum CsA concentration exceeding 1,000 ng/mL at 13 h post-ingestion, no CNS manifestations were observed, and the patient was discharged without adverse outcomes following therapeutic intervention.

**Table 1:** Summary of clinical manifestations and management strategies in pediatric cyclosporine A intoxication.

Date	Age	Underlying Disease	Dosage, (mg/kg)	Total dose (mg)	Route of administration	Concentration(ng/mL), time after dose	Signs and symptoms	Treatment	Prognosis
Arellano et al. 1991 (4)	2mo	Heart transplant	NS	240	Oral	NA	Hypotension, pallor, wheezing, tachycardia		Recover
	11y		NS	500	Oral	408, 12 h	Vomiting, hypertension	No treatment	Recover
	2.5y	Bone marrow transplant	NS	1,600	Oral	126, 24 h		No treatment	No adverse outcomes
	4y		40	400	Oral	NA		No treatment	unknown
	3y	Kidney transplant	45	600	Oral	NA	Asymptomatic	No treatment	No adverse outcomes
	3y	Liver transplant	47	600	Oral	459, 5 h	Asymptomatic	No treatment	No adverse outcomes
	2mo	Liver transplant	104	500	Oral	447, 6 h	Vomiting	No treatment	Recover
	11y	Bone marrow transplant	149	5,000	Oral	8,200, 8 h	Vomiting, mild drowsiness	No treatment	Recover
	1y	Nephrotic syndrome	20	NS	Oral	NA	Asymptomatic	No treatment	No adverse outcomes
	12d	Prematurity	179	100	IM	NA	Metabolic acidosis, cyanosis, worsened kidney function	Intubation, resuscitative measures, total blood exchange	Death
	Neonate	Thrombocytopenia	400	1,200	IV	5,288, a few hours 13,045, during transfusion	Cyanosis, metabolic acidosis, hypopnea, acute kidney failure, anemia	Total blood exchange	Recover
	44d	Prematurity, neuroblastoma	18.7	50	IM	520, 2 h	Oliguria, hyponatremia, edema	No treatment	Recover
	Neonate	Prematurity	NS	100	IM	NA	Hyponatremia	Intubation, resuscitative measures, transfusion	Recover
	8d	Prematurity	42.9	100	IM	2,040, 12 h 2,400, 21 h	Oliguria, hyponatremia	No treatment	Recover
	10d	Prematurity	49	100	IM	2,540, 24 h	Oliguria, hyponatremia	No treatment	Recover
Anderson et al. 1992 (18)	4.5y	Kidney transplant	170	3,000	Oral	190, 2 h	Intoxicated appearance	Emesis-inducing medicine, Activated charcoal plus sorbitol	Recover
Ceschi et al. 2013 (15)	4m	Unknown condition requiring immunosuppression	12	60	Oral	374, unknown	Asymptomatic	Dimethicone	No adverse outcomes
	3y	Kidney transplant	20.83	250	Oral	NA	Asymptomatic	No treatment	No adverse outcomes
	4y	Bone marrow transplant	16.7	300	Oral	1,500, immediately	Transient kidney impairment	No treatment	Recover
	2mo	Hemophagocytic lymphohistiocytosis	315.8	1,200	Oral	836, 15 h	Transient rise in systolic blood pressure	Nasogastric tube aspiration	Recover
	5mo	Heart transplant	50	300	Oral	750, 8 h	Nausea	Activated charcoal	Recover
	15y	Lung transplant	12.27	550	Oral	1,395, 3 h	Asymptomatic		No adverse outcomes
	16y	None (cohabitant of a CsA-user)	4.35	200	Oral	NA	Asymptomatic		No adverse outcomes
Diav-Citrin et al. 2000 (13)	1y	Immunodeficiency	70	5,000	Oral	NA	Irritability, face flushing, hypertension	No treatment	Recover
Gaggero et al. 2006 (17)	14y	Acute lymphoblastic leukemia			Oral	1,300, unknown	Seizure	Benzodiazepine	
	8y	Fanconi's anemia			Oral	768, unknown	Seizure, coma	Benzodiazepine	
	4y	Osteopetrosis			Oral	2,410, unknown	Seizure	Benzodiazepine	Epilepsy
	15y	Acute lymphoblastic leukemia			Oral	551, unknown	Seizure, coma	Benzodiazepine	
	9y	Acute lymphoblastic leukemia			Oral	1,824, unknown	Seizure, coma	Benzodiazepine	
Gulmez et al. 2025 (3)	2y	Nephrotic syndrome	15	250 q 12 h × 2	IV	2,605, unknown	Flushing, drowsiness	N-acetylcystein, rifampine, phenobarbital	Recover
Current case	3.9y	Nephrotic syndrome	75	1,500	Oral	1,003, 13h	Abdominal pain, diarrhea, vomiting	Rifampine, phenobarbital	Recover

Clinical experience regarding the management of acute CsA intoxication is limited (2, 3, 12, 14). Since CNIs are non-dialyzable and there is a lack of antidotes or effective elimination strategies, the primary therapy is to immediately stop the drug and provide supportive care. For oral administration, absorption of CsA from the gastrointestinal tract is reported to be slow and variable, with peak concentration occurring from 1 to 8 h after dosing (2). Thus, gastrointestinal decontamination methods, including gastric lavage, induced vomiting with ipecac syrup, and activated charcoal with sorbitol, can aid oral overdose (4, 18). Ershad et al. suggest that charcoal administration may be effective 5 h or more after an overdose in certain patients (2, 19). In addition, the use of CYP3A4 inducers such as phenobarbital, phenytoin, and rifampicin is a potential treatment option to accelerate the clearance of CNIs (11, 12). By enhancing CsA metabolism, these agents effectively reduce its blood concentrations. Given that neurotoxicity is the most prominent acute symptom of CNIs toxicity, the use of anticonvulsants such as phenytoin and phenobarbital appear to have more benefits than risks. Because the longer half-life and exclusive renal elimination of phenobarbital may increase risk of adverse drug reaction, phenytoin is more commonly the preferred CYP inducer in majority of cases (12). However, some reports have described that phenytoin, owing to its higher affinity for plasma protein compared with phenobarbital, carries the theoretical risk of competing with CNIs and exacerbating toxicity (20). Based on this consideration, we used phenobarbital in our patient. Additionally, rifampicin was added into the treatment regimen, taking advantage of its favorable pharmacokinetic properties, including a shorter half-life, potent induction of both CYP enzymes and intestinal P-glycoprotein, and a predominantly non-renal elimination (12). While various strategies using phenobarbital, phenytoin, and rifampicin have been documented in 34 clinical reports, there is a notable lack of studies comparing isolated CNI withdrawal and active pharmacological induction. In practice, a tailored, patient-centric approach is essential when selecting the induction agent and its administration route. To ensure patient safety, clinicians should implement stringent therapeutic drug monitoring of CNI levels and limit the duration of enzyme-inducing therapy to the minimum necessary period, typically three to four days (12). Additional treatments such as extracorporeal elimination, plasmapheresis, and whole blood exchange have uncertain and minimal benefits in children (2, 4). In our case, rifampin and phenobarbital were used. Following two doses of phenobarbital, the drug concentration dropped below 500, threshold associated with seizure, allowing the discontinuation of the medication. Despite the absence of pharmacodynamic assessments of CsA due to the 12-hour delay in reporting the overdose, the consistent decline in CsA concentrations toward therapeutic ranges without any complications could be contributed to the accelerated systemic clearance facilitated by CYP induction therapy.

This case report has some limitations to consider. Due to the 13-hour delay between the medication error and hospital arrival, we were unable to measure the absolute peak plasma CsA concentration or observe the patient's clinical status during the first few hours of the overdose. Additionally, as this is a single case report, the favorable outcome observed here may not be generalizable to all pediatric patients, particularly those with different underlying comorbidities or those who ingest even higher doses. Despite these limitations, this case holds significant clinical strength as it demonstrates the successful management of a massive CsA overdose in a pediatric patient, even with a delayed diagnosis. This underscores the critical importance of immediate diagnostic recognition followed by intensive observation and proactive management in mitigating the risks of severe accidental toxicity.

In conclusion, while pediatric patients remain vulnerable to cyclosporine overdose, acute overdose may not always lead to severe or life-threatening outcomes. However, early and effective management is critical to prevent serious adverse events.

## Data Availability

The original contributions presented in the study are included in the article/Supplementary Material, further inquiries can be directed to the corresponding author.
