# Correction: Acute cyclosporine overdose in a child with nephrotic syndrome: a case report and literature review

**DOI:** 10.3389/fped.2026.1838282

**Published:** 2026-04-15

**Authors:** Eun Song Song, Eun Mi Yang

**Affiliations:** Department of Pediatrics, Chonnam National University Hospital and Medical School, Gwangju, Republic of Korea

**Keywords:** acute toxicity, children, cyclosporine, nephrotic syndrome, treatment

The funding from Chonnam National University was displayed as “2025-0375-01”. The correct statement is “Chonnam National University (Grant number: 2025-0372-01)”

The original version of this article has been updated.

